# A Clinically Applicable Nomogram for Predicting the Risk of Invasive Mechanical Ventilation in *Pneumocystis jirovecii* Pneumonia

**DOI:** 10.3389/fcimb.2022.850741

**Published:** 2022-03-10

**Authors:** Rongjun Wan, Lu Bai, Yusheng Yan, Jianmin Li, Qingkai Luo, Hua Huang, Lingmei Huang, Zhi Xiang, Qing Luo, Zi Gu, Qing Guo, Pinhua Pan, Rongli Lu, Yimin Fang, Chengping Hu, Juan Jiang, Yuanyuan Li

**Affiliations:** ^1^Department of Respiratory Medicine, National Key Clinical Specialty, Branch of National Clinical Research Center for Respiratory Disease, Xiangya Hospital, Central South University, Changsha, China; ^2^Center of Respiratory Medicine, Xiangya Hospital, Central South University, Changsha, China; ^3^Clinical Research Center for Respiratory Diseases in Hunan Province, Changsha, China; ^4^Hunan Engineering Research Center for Intelligent Diagnosis and Treatment of Respiratory Disease, Changsha, China; ^5^National Clinical Research Center for Geriatric Disorders, Xiangya Hospital, Changsha, China; ^6^Department of Pulmonary and Critical Care Medicine, First Hospital of Changsha, Changsha, China; ^7^Department of Pulmonary and Critical Care Medicine, Hunan Provincial People’s Hospital, First Affiliated Hospital of Hunan Normal University, Changsha, China; ^8^Department of Pulmonary and Critical Care Medicine, First People’s Hospital of Chenzhou, Chenzhou, China; ^9^Medical Center of Tuberculosis, Second People’s Hospital of Chenzhou, Chenzhou, China; ^10^Department of Pulmonary and Critical Care Medicine, Yueyang Central Hospital, Yueyang, China; ^11^Department of Respiratory Medicine, First People’s Hospital of Huaihua, Huaihua, China; ^12^Department of Pulmonary and Critical Care Medicine, Second Affiliated Hospital, Hengyang Medical School, University of South China, Hengyang, China; ^13^Department of Pulmonary and Critical Care Medicine, Xiangtan Central Hospital, Xiangtan, China; ^14^Department of Pulmonary and Critical Care Medicine, Yiyang Central Hospital, Yiyang, China

**Keywords:** *Pneumocystis jirovecii* pneumonia (PCP), invasive mechanical ventilation (IMV), predictive model, nomogram, machine learning

## Abstract

**Objective:**

*Pneumocystis jirovecii* pneumonia (PCP) is a life-threatening disease associated with a high mortality rate among immunocompromised patient populations. Invasive mechanical ventilation (IMV) is a crucial component of treatment for PCP patients with progressive hypoxemia. This study explored the risk factors for IMV and established a model for early predicting the risk of IMV among patients with PCP.

**Methods:**

A multicenter, observational cohort study was conducted in 10 hospitals in China. Patients diagnosed with PCP were included, and their baseline clinical characteristics were collected. A Boruta analysis was performed to identify potentially important clinical features associated with the use of IMV during hospitalization. Selected variables were further analyzed using univariate and multivariable logistic regression. A logistic regression model was established based on independent risk factors for IMV and visualized using a nomogram.

**Results:**

In total, 103 patients comprised the training cohort for model development, and 45 comprised the validation cohort to confirm the model’s performance. No significant differences were observed in baseline clinical characteristics between the training and validation cohorts. Boruta analysis identified eight clinical features associated with IMV, three of which were further confirmed to be independent risk factors for IMV, including age (odds ratio [OR] 2.615 [95% confidence interval (CI) 1.110–6.159]; *p* = 0.028), oxygenation index (OR 0.217 [95% CI 0.078–0.604]; *p* = 0.003), and serum lactate dehydrogenase level (OR 1.864 [95% CI 1.040–3.341]; *p* = 0.037). Incorporating these three variables, the nomogram achieved good concordance indices of 0.829 (95% CI 0.752–0.906) and 0.818 (95% CI 0.686–0.950) in predicting IMV in the training and validation cohorts, respectively, and had well-fitted calibration curves.

**Conclusions:**

The nomogram demonstrated accurate prediction of IMV in patients with PCP. Clinical application of this model enables early identification of patients with PCP who require IMV, which, in turn, may lead to rational therapeutic choices and improved clinical outcomes.

## Introduction

*Pneumocystis jirovecii* pneumonia (PCP) is an opportunistic fungal infection that commonly affects immunocompromised patient populations. It remains a severe disease associated with a high mortality rate among patients either positive or negative for human immunodeficiency virus (HIV) infection (Roux et al., 2014; Alanio et al., 2016). While the incidence of PCP among HIV-positive patients has declined with the widespread use of antiretroviral and prophylactic therapy (Morris et al., 2004; Salzer et al., 2018), PCP has become more frequent in HIV-negative populations in recent years due to the increasing prevalence of immunosuppressive conditions such as hematological malignancies, solid tumors, systemic corticosteroid and immunosuppressive therapies, organ transplantation, and hematopoietic stem cell transplantation (Jin et al., 2021; Wang et al., 2021). PCP has been reported to be associated with mortality rates as high as 10%–20% in HIV and 30%–60% in non-HIV patients (Cilloniz et al., 2019).

Patients with PCP are usually characterized by acute or subacute hypoxemia and exhibit symptoms of progressive dyspnea. Adherence of *Pneumocystis* to the alveoli triggers a severe inflammatory response in hosts that causes diffuse lung injury and impairs gas exchange (Hahn and Limper, 2003). In a substantial proportion of cases, hypoxemia can progress to respiratory failure and even acute respiratory distress syndrome within a short period of time. Mechanical ventilation, including invasive mechanical ventilation (IMV), is a crucial component of the clinical management of patients with PCP. According to data from previous studies, 16% of HIV-infected and 50%–64% of non-HIV-infected PCP patients require mechanical ventilation during hospitalization (Nüesch et al., 1999; Kim et al., 2014). A large cohort study also reported that >40% of patients required admission to the intensive care unit, and approximately 37% required mechanical ventilation (Schmidt et al., 2018). Approximately 28% of patients with PCP require IMV. In particular, the use of IMV is more frequent (56.6%) in patients with severe PCP (Gaborit et al., 2019). For patients who require mechanical ventilation, mortality can be as high as 62% (Monnet et al., 2008). Late intubation has been recognized to be associated with increased mortality in those with acute respiratory distress syndrome (Kangelaris et al., 2016). Furthermore, it has been found that failure of non-invasive mechanical ventilation is significantly associated with hospital survival among PCP patients, with a mortality rate as high as 88.9% (Wang et al., 2020). Therefore, early identification of patients who require IMV can provide guidance for prompt initiation of IMV and optimize the allocation of medical resources. However, while prognostic factors for mortality have been well established (Walzer et al., 2008; Fei et al., 2009; Wang et al., 2011; Weng et al., 2016; Gaborit et al., 2019), little is known about the early prediction of requirements for IMV in patients with PCP so far.

Herein, we developed a visual and easy-to-use nomogram to predict the risk for IMV in PCP patients on admission to the hospital using a machine learning method.

## Methods

### Study Design and Patients

The present multicenter, observational cohort study collected clinical data from all immunosuppressed patients who were diagnosed with PCP between January 1, 2018, and October 31, 2021, in 10 tertiary care hospitals in China. Clinical data included demographic information, clinical manifestations, comorbidities, laboratory indicators, vital signs, anti-*Pneumocystis jirovecii* treatment and respiratory support approaches. All baseline laboratory tests were performed within 24 h of patient admission. Adult patients (age ≥ 18 years) were eligible for enrollment if they fulfilled all of the following criteria: immunosuppressed conditions, including HIV-positive and HIV-negative patients (Ramirez et al., 2020); typical clinical manifestations of PCP, including fever, cough (usually dry cough), and progressive dyspnea; radiological findings suggestive of PCP in the bilateral lungs that newly emerged on chest computed tomography; and a positive result for *Pneumocystis jirovecii* by Gomori methenamine silver staining, real-time polymerase chain reaction assay, and/or metagenomic next-generation sequencing (Jiang et al., 2021). Diagnosis of PCP was confirmed after consultation with ≥ 2 senior pulmonary specialists at each research center, based on clinical symptoms, laboratory findings, chest radiology, microbiological tests, and treatment response. Patients < 18 years of age, those who were pregnant, and those with incomplete medical records were excluded from the study. Eligible patients, who were diagnosed with PCP between January 1, 2018, and November 30, 2020, were included in the training cohort. Clinical data from these patients were retrospectively collected for the development of a nomogram. Patients who were diagnosed with PCP between January 1, 2021, and October 31, 2021, were included in the validation cohort. Clinical data from these patients were prospectively collected to validate the nomogram.

### Relevant Variable Identification Using Random Forest

To identify clinical features that had a larger than random association with IMV use, Boruta analysis was performed (Kursa and Rudnicki, 2010) to cover all data regarding selected variables obtained when patients were admitted to hospital. The Boruta analysis is a random forest-based feature selection algorithm (Breiman, 2001) that performs multiple runs of random forest to compare shuffled random variables to the original variables. At the same time, scores representing importance are assigned to each variable. All analyzed variables were divided into rejected, tentative, and accepted groups based on their importance scores. Briefly, variables with importance scores below the shadowMax value are rejected by Boruta, while those above are accepted; other variables with importance scores around shadowMax value, which cannot be rejected or accepted, are divided into a tentative group. All Boruta analyses were performed using the Boruta package (Kursa and Rudnicki, 2010) version 6.0.0 using the default parameters (ntree = 500, maxRuns = 100, *p* = 0.01). After the Boruta analysis, the accepted variables were further analyzed using univariable and multivariable logistic regression to calculate odds ratio (OR) and corresponding 95% confidence interval (CI) to confirm whether the selected variables are risk factors for IMV in patients with PCP.

### Development and Validation of a Logistic Regression Model

To develop a practical and easy-to-use predictive model for IMV using clinical data acquired on admission, clinical data from all enrolled patients in the training cohort (*n* = 103) were analyzed to start the establishment procedure (**Table 1**). Important variables identified by the Boruta algorithm were further analyzed using univariable logistic regression to calculate the OR and to assess significance in the training cohort. Variables that were statistically significant were further included in the multivariable logistic regression analysis to confirm whether they were independent risk factors. Subsequently, a logistic regression model was established based on factors with statistical significance in multivariable logistic regression and visualized using a nomogram. Briefly, the nomogram is based on proportionally converting each regression coefficient in multivariate logistic regression analysis to a 0- to 100-point scale. The effect of the variable with the highest *β* coefficient (absolute value) was assigned 100 points. The points are added across independent variables to obtain the total points, which are converted to predicted probabilities (Lei et al., 2016). Furthermore, the model was evaluated in a validation cohort (*n* = 45), for which the number of patients was determined based on the ratio of the samples in the training and validation sets recommended in previous studies (Frizzell et al., 2017; Huang et al., 2018). Predictive performance was assessed by discrimination and calibration.

**Table 1:** Baseline characteristics of patients.

Variables [*n* (%), median (IQR) or mean ± SD]	Overall (*n* = 148)	Training cohort (*n* = 103)	Validation cohort (*n* = 45)	*p*-value
Age, years	51.0 ± 15.2	50.5 ± 16.1	52.0 ± 13.1	0.573
Gender				
Male	98 (66.2)	70 (68.0)	28 (62.2)	
Female	50 (33.8)	33 (32.0)	17 (37.8)	0.624
Comorbidity				
AIDS	45 (30.4)	36 (35.0)	9 (20.0)	0.104
Rheumatic diseases	38 (25.7)	22 (21.4)	16 (35.6)	0.107
Solid tumors	13 (8.8)	6 (5.8)	7 (15.6)	0.065
Hematological tumors	16 (10.8)	12 (11.7)	4 (8.9)	0.777
Organ transplantation	8 (5.4)	6 (5.8)	2 (4.4)	1.000
ILD	10 (6.8)	4 (3.9)	6 (13.3)	0.068
PTB	6 (4.1)	5 (4.9)	1 (2.2)	0.668
COPD	9 (6.1)	6 (5.8)	3 (6.7)	1.000
Asthma	1 (0.7)	1 (1.0)	0	1.000
OI (mmHg)	217.5 (149.5, 326.3)	228.0 (164.5, 330.0)	188.0 (141.0, 288.0)	0.103
WBC (×10^9^/L)	7.1 (5.2, 10.2)	6.9 (5.0, 9.5)	8.0 (5.7, 11.1)	0.076
Neu (×10^9^/L)	5.8 (3.7, 9.1)	5.5 (3.6, 8.5)	6.7 (4.7, 10.1)	0.060
Lym (×10^9^/L)	0.7 (0.4, 1.0)	0.7 (0.5, 1.0)	0.6 (0.4, 0.9)	0.459
Hemoglobin (g/L)	104.0 ± 22.4	103.5 ± 22.9	105.2 ± 21.5	0.677
Platelet (×10^9^/L)	197.0 ± 105.5	185.5 ± 103.6	223.1 ± 106.2	0.046
PCT (ng/ml)	0.21 (0.09, 0.78)	0.21 (0.10, 0.60)	0.27 (0.08, 1.10)	0.616
CRP (mg/L)	76.4 (37.5, 119.9)	67.1 (38.8, 104.9)	94.6 (24.3, 182.0)	0.089
Serum BDG (ng/L)	139.4 (38.1, 370.7)	186.4 (54.3, 449.7)	89.0 (37.5, 254.7)	0.127
Serum GM (ng/L)	0.25 (0.18, 0.41)	0.29 (0.19, 0.56)	0.24 (0.18, 0.34)	0.106
LDH (U/L)	425.7 (318.5, 653.3)	422.0 (304.5, 681.5)	442.0 (354.0, 501.8)	0.820
Albumin (g/L)	28.4 ± 6.4	28.6 ± 6.6	28.0 ± 6.0	0.633
Globulin (g/L)	27.3 (22.5, 34.5)	27.6 (22.6, 34.7)	26.8 (22.2, 33.7)	0.470
TBil (μmol/L)	7.9 (5.4, 11.5)	7.9 (5.4, 11.8)	7.4 (5.4, 11.2)	0.687
SCr (μmol/L)	68.0 (52.6, 95.3)	67.9 (52.7, 92.9)	68.3 (51.8, 106.0)	0.650
BUN (mmol/L)	6.0 (4.1, 9.8)	5.7 (3.7, 9.7)	6.4 (4.3, 11.9)	0.187
Vital signs				
Heart rate (bpm)	102 ± 19	101 ± 20	104 ± 19	0.264
Respiratory rate (bpm)	22 (20–26)	22 (20–25)	23 (21–30)	0.040
SpO_2_ (%)	95 (90–97)	95 (91–97)	94 (89–97)	0.699
SBP (mmHg)	115 (100–126)	112 (100–125)	118 (100–130)	0.403
DBP (mmHg)	70 (63–80)	71 (63–80)	71 (63–78)	0.657
Co-infections				
Viruses	44 (30.1)	29 (28.4)	15 (34.1)	0.626
Bacteria	83 (56.8)	58 (56.9)	25 (56.8)	1.000
Other fungi	17 (11.5)	13 (12.6)	4 (8.9)	0.708
Anti-PJ treatment				
TMP-SMZ only	71 (48.0)	53 (51.5)	18 (40.0)	0.506
TMP-SMZ + echinocandins	67 (45.3)	44 (42.7)	23 (51.1)	
Days from admission to anti-PJ treatment	1 (0–4)	1 (0–4)	1 (0–2.5)	0.368
Systemic use of glucocorticoids	117 (79.1)	78 (75.7)	39 (86.7)	0.199
Use of IMV	68 (45.9)	45 (43.7)	23 (51.1)	0.513

IQR, interquartile ranges; SD, standard deviation; AIDS, acquired immune deficiency syndrome; PTB, pulmonary tuberculosis; COPD, chronic obstructive pulmonary disease; ILD, interstitial lung disease; OI, oxygenation index; WBC, white blood cell; Neu, neutrophil; Lym, lymphocyte; PCT, procalcitonin; CRP, C-reaction protein; BDG, (1,3)-β-D-glucan; GM, galactomannan; LDH, lactate dehydrogenase; TBil, total bilirubin; SCr, serum creatine; BUN, blood urea nitrogen; bpm, beats or breaths per minute; SpO_2_, the percent saturation of oxygen in the blood; SBP, systolic blood pressure; DBP, diastolic blood pressure; PJ, Pneumocystis jirovecii; TMP-SMZ, trimethoprim-sulfamethoxazole; IMV, invasive mechanical ventilation.

### Statistical Analysis

All continuous variables were tested for normal distribution using the Lilliefors test (Lilliefors, 1967) before statistical expression and comparison. Normally distributed continuous variables are expressed as mean ± standard deviation (SD) and compared using Student’s *t*-test, while non-normally distributed continuous variables are expressed as median (interquartile range) and were compared using the Mann–Whitney *U* test. Categorical variables are expressed as count and percentage and were compared using the chi-squared or Fisher’s exact tests.

Important variables selected by the Boruta algorithm were successively included in the univariate and multivariate logistic regression analyses, and a clinical predictive model was constructed using multivariable logistic regression. Furthermore, the discriminative ability of the model was assessed using receiver operator characteristic (ROC) curve and area under the ROC curve (AUROC), which was assessed using a bootstrap-based calibration process (Steyerberg and Vergouwe, 2014; Lindhiem et al., 2020) and Spiegelhalter’s z test (Spiegelhalter, 1986) to evaluate the relationship between the actual and predicted probability of IMV. A non-significant result in the Spiegelhalter’s z-test indicated that the model was well fitted. These analyses were performed using the rms version 5.1 and pROC version 1.16.2 R packages.

All statistical analyses were performed using R software version 3.6.3 (www.r-project.org). Except for the nomogram, figures were plotted using ggplot2 version 3.3.2 R package. For all analyses, differences with *p* < 0.05 were considered to be statistically significant.

### Ethics Statement

The present study was approved by the Ethics Committee of Xiangya Hospital, Central South University (Changsha, Hunan, China; 202112655). Due to the use of de-identified and anonymized research data, requirements for informed written consent were waived. The study was conducted in accordance with the Declaration of Helsinki.

## Results

### Baseline Characteristics of Patients

A total of 148 PCP patients who met the inclusion criteria were enrolled in this study. Of these, 103 patients were included into the training cohort, and 45 were in validation cohort. Baseline clinical characteristics are shown in **Table 1**. The average age of all patients was 51 years old, and 66.2% of them were men. The most common comorbidities included AIDS (30.4%), rheumatic diseases (25.7%), and hematological malignancies (10.8%). Marked laboratory abnormalities were observed, including decreased oxygenation index (the ratio of PaO_2_ to FiO_2_), lymphocytopenia, elevated serum levels of (1,3)-β-D-glucan, and lactate dehydrogenase (LDH). Co-infections were common in patients with PCP. Bacterial and viral co-infections were found in 56.8% and 30.1% of patients, respectively. Among all patients, 48.0% received TMP-SMZ only, while 45.3% received TMP-SMZ combined with echinocandins. The median time from admission to initiation of anti-*Pneumocystis jirovecii* treatment was 1 day. Systemic glucocorticoids was used in 79.1% of patients. Overall, there was no significant difference in baseline clinical characteristics between training and validation cohorts. IMV was used in 45 (43.7%) and 23 (51.1%) patients in these two cohorts, respectively.

### Identification of Important Variables by Boruta Analysis

**Figure 1:**
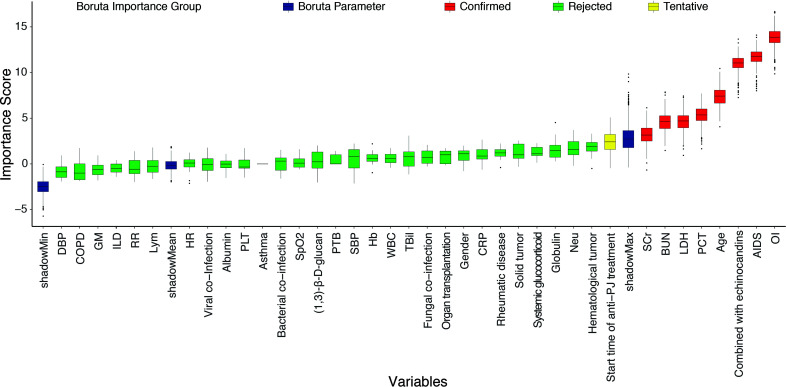
The feature importance in the Boruta feature selection process. The red box showed the features that are confirmed as important, the green box showed the features confirmed as unimportant, the yellow box showed the features that are tentative, and the blue box showed Boruta parameters. AIDS, acquired immune deficiency syndrome; PTB, pulmonary tuberculosis; COPD, chronic obstructive pulmonary disease; ILD, interstitial lung disease; OI, oxygenation index; WBC, white blood cell; Neu, neutrophil; Lym, lymphocyte; Hb, hemoglobin; PLT, platelet; PCT, procalcitonin; CRP, C-reaction protein; GM, galactomannan; LDH, lactate dehydrogenase; TBil, total bilirubin; SCr, serum creatine; BUN, blood urea nitrogen; HR, heart rate; RR, respiratory rate; SpO_2_, the percent saturation of oxygen in the blood; SBP, systolic blood pressure; DBP, diastolic blood pressure.

Based on the baseline characteristics of all patients, 38 clinical features were evaluated by using Boruta feature selection method. The rank plot of feature importance is shown in **Figure 1**. There were 8 clinical features confirmed as important, including oxygenation index, AIDS, combination with echinocandins, age, serum LDH, procalcitonin, serum creatinine, and blood urea nitrogen. Unsurprisingly, baseline oxygenation index had the highest importance associated with the risk of IMV. The other variables with an importance score lower than shadowMax were all confirmed as unimportant.

### Development and Validation of a Predictive Nomogram of IMV

**Figure 2:**
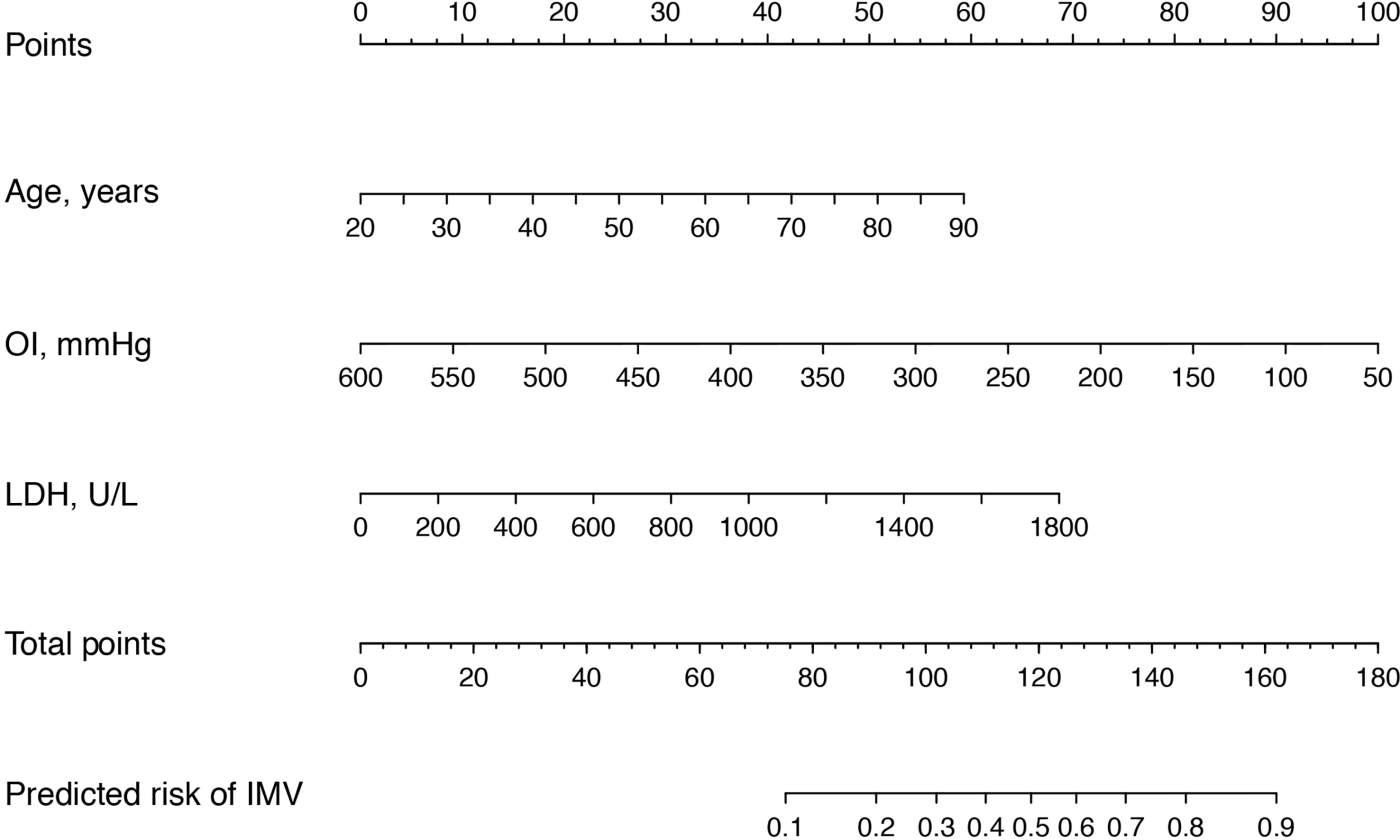
Nomogram for the estimation of invasive mechanical ventilation risk in PCP patients. To use the nomogram, find the position of each variable on the corresponding axis, and draw a line to the points axis (top) for the number of points. Then, sum up the points from all of the variables, and draw a line from the total points axis to the lower line of the nomogram to determine the predicted risk of invasive mechanical ventilation. OI, oxygenation index; LDH, lactate dehydrogenase.

Next, these 8 important variables selected by Boruta were further analyzed by univariate logistic regression, and the results are presented in **Table 2**. There were 5 variables associated with the risk of IMV, including age, AIDS, oxygenation index, serum LDH, and blood urea nitrogen. In subsequent multivariable logistic regression, age (OR, 2.615; 95% CI, 1.110–6.159; *p* = 0.028), oxygenation index (OR, 0.217; 95% CI, 0.078–0.604; *p* = 0.003), and serum LDH (OR, 1.864; 95% CI, 1.040-3.341; *p* = 0.037) were confirmed as independently associated with the risk of IMV in PCP patients (**Table 3**). These factors were used to construct a predictive model for IMV risk by multivariable logistic regression, which was further visualized by nomogram as shown in **Figure 2**.

**Table 2:** Univariate logistic regression analysis of invasive mechanical ventilation based on baseline characteristics in the training cohort.

Variables	OR	95% CI	*p-*value
Age	2.556	1.324–4.932	0.005
AIDS	0.184	0.071–0.479	0.001
OI	0.173	0.078–0.388	<0.001
LDH	2.235	1.347–3.707	0.002
BUN	1.632	1.046–2.547	0.031
SCr	1.115	0.929–1.338	0.242
PCT	1.002	0.984–1.020	0.846
Combined with echinocandins	4.424	1.871–10.465	0.659

OR, odds ratio; CI, confidence interval; AIDS, acquired immunodeficiency syndrome; OI, oxygenation index; PCT, procalcitonin; LDH, lactate dehydrogenase; SCr, serum creatine; BUN, blood urea nitrogen.

**Table 3:** Multivariate logistic regression analysis of invasive mechanical ventilation based on baseline characteristics in the training cohort.

Variables	OR	95% CI	*p-*value
Age	2.615	1.110–6.159	0.028
AIDS	0.930	0.238–3.625	0.916
OI	0.217	0.078–0.604	0.003
LDH	1.864	1.040–3.341	0.037
BUN	1.181	0.701–1.989	0.531

OR, odds ratio; CI, confidence interval; AIDS, acquired immunodeficiency syndrome; OI, oxygenation index; LDH, lactate dehydrogenase; BUN, blood urea nitrogen.

**Figure 3:**
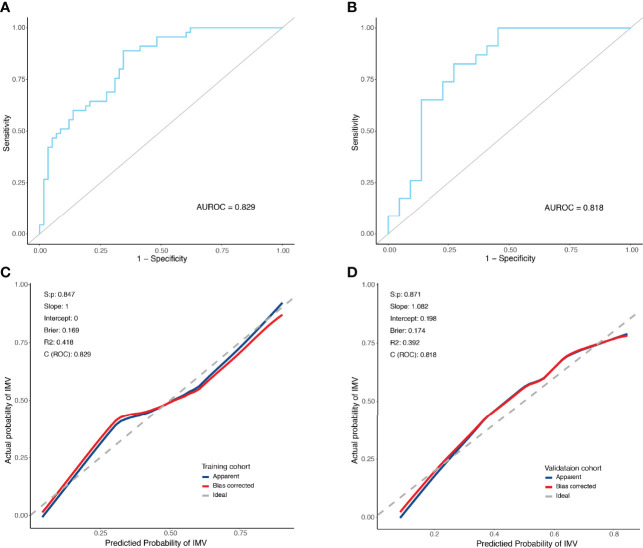
Receiver operating characteristic curves and predictive performance of the nomogram for invasive mechanical ventilation in PCP patients. **(A)** ROC curve for the predictive model of the training cohort. Area under the curve was 0.829. **(B)** ROC curve for the predictive model of the validation cohort. Area under the curve was 0.818. **(C)** Validity of the predictive performance of the nomogram in estimating the risk of invasive mechanical ventilation in the training cohort. **(D)** Validity of the predictive performance of the nomogram in estimating the risk of invasive mechanical ventilation in the validation cohort. C index, concordance index; ROC, receiver operating characteristic.

The predictive model was internally validated using the bootstrap validation method. Receiver operating characteristic curves for the predictive model of training and validation cohorts are shown in **Figures 3A, B**. The nomogram demonstrated good accuracy in estimating the risk of IMV, with a bootstrap-corrected C index of 0.829 (95% CI, 0.752–0.906). Furthermore, calibration plots graphically showed good agreement between the estimated risk by the nomogram and the actual use of IMV in PCP patients (**Figure 3C**). Next, we applied our model to the validation cohort and performed relative analysis to evaluate the performance of nomogram. the results displayed a C index of 0.818 (95% CI, 0.686–0.950) for the estimation of IMV risk. There was also a good calibration curve for the risk estimation (**Figure 3D**). The sensitivity, specificity, positive predictive value, and negative predictive value of this nomogram in identifying the patients who require IMV were 88.9%, 65.5%, 66.7%, and 88.4% in the training cohort, and 82.6%, 72.7%, 76.0%, and 80.0% in the validation cohort, respectively.

## Discussion

PCP is a life-threatening disease associated with the frequent use of IMV in immunocompromised patient populations. In this multicenter study, we designed and developed an easy-to-use visual nomogram to predict the risk for IMV in patients with PCP. The nomogram demonstrated good predictive ability for IMV using three common patient variables collected on admission; more specifically, age, oxygenation index, and serum LDH level. To the best of our knowledge, this is the first study to report a clinically applicable predictive model for the early estimation of IMV risk in patients with PCP.

In this study, our data demonstrated that oxygenation index was the most important risk factor for IMV. It is well recognized that the severity of PCP is determined based on the partial pressure of oxygen (*Pa*O_2_) while breathing room air and at rest. Patients were classified as having mild (*Pa*O_2_ > 70 mmHg), moderate (60 mmHg ≤ *Pa*O_2_ ≤ 70 mmHg), or severe (*Pa*O_2_ < 60 mmHg) PCP (Moon et al., 2011). A low oxygenation index reflects severe hypoxemia, which conceivably increases the risk for disease deterioration and requirement for IMV. Previous studies have demonstrated that poor oxygenation on admission to the hospital is a risk factor for mortality among HIV-infected patients (Walzer et al., 2008; Fei et al., 2009). Moreover, it has also been reported that age is independently associated with disease severity and mortality in patients with PCP. In a prospective observational study that enrolled 107 patients with PCP, Gaborit et al. reported that older age (OR 3.36 [95% CI 1.4–8.5]) was independently associated with disease severity (Gaborit et al., 2019). Another bicentric retrospective study also suggested that age (OR 1.051 [95% CI 1.007–1.097]) was an independent risk factor for hospital mortality for non-HIV patients in the intensive care unit, although its effect was only slight. Old age has been consistently correlated with worse prognosis in patients with connective tissue disease-associated PCP (Ishikawa et al., 2021). In the present study, multivariate logistic regression analysis confirmed that age was independently associated with the risk for IMV (OR 2.615 [95% CI 1.110–6.159]), indicating that older PCP patients are more likely to require IMV during hospitalization. Together with previous studies, our data support that close attention should be devoted to elderly patients with PCP.

LDH has long been recognized as a useful but non-specific serological marker in the diagnosis of PCP (White et al., 2017). Serum LDH levels can reflect the severity of radiographic abnormalities in PCP (Boldt and Bai, 1997). Schmidt et al. found that baseline serum LDH level was a predictor of in-hospital mortality (OR 1.17 [95% CI 1.09–1.27]) (Schmidt et al., 2018). However, there is no established association between serum LDH level and the risk for IMV in PCP. Interestingly, our data revealed that serum LDH level was an independent risk factor for IMV during hospitalization (OR 1.864 [95% CI 1.040–3.341]). This can be explained by the hypothesis that extracellular LDH levels indicate cell damage or cell death, and elevated serum LDH level is correlated with lung tissue damage. In fact, serum LDH level has been reported to be significantly higher (9.79 ± 7.0 versus 5.93 ± 3.9 μkat/L) among patients with severe PCP compared to those with non-severe PCP (Gaborit et al., 2019), which supports the hypothesis proposed above.

Based on our data, it is noteworthy that HIV infection was not independently associated with the risk for IMV among patients with PCP. It is well known that HIV-negative PCP patients usually experience a more progressive disease course, more severe hypoxemia, and a higher mortality rate (Roux et al., 2014; Cilloniz et al., 2019; Ghembaza et al., 2020). In the present study, HIV infection appeared to be associated with IMV use according to univariate logistic regression analysis. However, no statistical significance was observed in the multivariate logistic regression analysis. The effect of confounding factors can be an important reason, including oxygenation index, age, serum level of LDH, and blood urea nitrogen. It has been known that clinical characteristics of HIV-positive and HIV-negative PCP patients are markedly different. Overall, HIV-positive PCP patients tend to be younger and have higher oxygenation index, lower serum LDH, and lower blood urea nitrogen levels (Mundo et al., 2020; Feng et al., 2021). Therefore, these variables, which reflect the severity of disease, can be confounding factors for AIDS/HIV infection in multivariate logistic regression analysis. Our results indicate that HIV infection itself did not directly affect the risk for IMV, even though disease severity is more prominent in HIV-negative PCP patients.

The use of a nomogram for estimating the risk for IMV in patients with PCP is a new concept. Our predictive model is visual and easy to use, enabling early and accurate identification of patients with PCP who require IMV before clinical decompensation. By calculating the total points based on three easily accessible clinical variables (i.e., age, oxygenation index, and serum LDH level), physicians can predict the risk for IMV for HIV-positive and HIV-negative PCP patients on admission to hospital in either urban or rural settings. More importantly, this model demonstrated good predictive performance, with C indexes of 0.829 (95% CI 0.752–0.906) and 0.818 (95% CI 0.686–0.950) in predicting IMV in the training and validation cohorts. In addition, the calibration curves were well fitted. The use of this predictive model could provide important guidance for clinical decision-making and optimizing the allocation of medical resources, with the hope of improving patient outcomes and quality of care. Meanwhile, it is of great significance to apply this predictive model in primary hospitals, where IMV is usually not possible. Physicians can easily estimate the risk of IMV once patients are admitted, which is useful in deciding whether to transfer them to higher-level hospitals. By using this predictive model, early and accurate identification of patients who are likely to require IMV will enable closer monitoring for signs and symptoms of clinical deterioration and optimize allocation of medical resources, which can facilitate clinical decision-making and may potentially improve the clinical outcomes of patients with PCP.

There were several limitations to the present study. First, clinical data from patients in the training cohort were retrospectively collected; thus, inherent bias was unavoidable. Second, although 10 tertiary care hospitals participated in this study, the sample size was relatively small, which may have affected the validity of our predictive model. Finally, whether this predictive model will improve the clinical outcomes of patients with PCP by enabling early identification of patients who require IMV warrants further evaluation in large-scale prospective studies. Nevertheless, we believe that this visual predictive model represents a useful tool for clinical physicians who are focused on PCP—especially severe PCP—but needs confirmation in future clinical trials.

## Conclusion

We developed a clinically applicable nomogram for predicting the risk for IMV in patients with PCP. This nomogram enables early and accurate estimation of IMV risk when PCP patients are admitted to the hospital, which may provide guidance for clinical decision-making.

## Data Availability Statement

Data are available related to this study can be accessible from the corresponding authors with reasonable requests.

## Ethics Statement

The present study was approved by the Ethics Committee of Xiangya Hospital, Central South University (Changsha, Hunan, China; 202112655). Due to the use of deidentified and anonymized research data, requirements for informed written consent were waived. The study was conducted in accordance with the Declaration of Helsinki.

## Author Contributions

Study concept and design: YL and JJ. Acquisition of data: YY, JL, LB, RW, QKL, HH, LH, ZX, QL, ZG, QG, PP, RL, YF, and CH. Statistical analysis: RW and LB. Analysis and interpretation of data: YL, JJ, RW, LB, YY, and JL. Drafting of the manuscript: JJ, LB, and RW. Critical revision of the manuscript: YL and JJ. All authors contributed to the article and approved the submitted version.

## Funding

This work was supported by grants from the National Natural Science Foundation of China (82170041, 82100099, and 81873406), the Innovative Research Platform of Hunan Development and Reform Commission (2021-212), and the Youth Research Foundation of Xiangya Hospital (2018Q015).

## Conflict of Interest

The authors declare that the research was conducted in the absence of any commercial or financial relationships that could be construed as a potential conflict of interest.

## Publisher’s Note

All claims expressed in this article are solely those of the authors and do not necessarily represent those of their affiliated organizations, or those of the publisher, the editors and the reviewers. Any product that may be evaluated in this article, or claim that may be made by its manufacturer, is not guaranteed or endorsed by the publisher.
